# Association between polymorphisms in *NOBOX* and litter size traits in Xiangsu pigs

**DOI:** 10.3389/fvets.2024.1359312

**Published:** 2024-03-08

**Authors:** Jiajin Huang, Yong Ruan, Meimei Xiao, Lingang Dai, Chuanmei Jiang, Jifeng Li, Jiali Xu, Xiang Chen, Houqiang Xu

**Affiliations:** ^1^Key Laboratory of Animal Genetics, Breeding and Reproduction in the Plateau Mountainous Region, Ministry of Education, Guizhou University, Guiyang, China; ^2^Guizhou Provincial Key Laboratory of Animal Genetics, Breeding and Reproduction, Guizhou University, Guiyang, China; ^3^College of Animal Science, Guizhou University, Guiyang, China

**Keywords:** *NOBOX* gene, pig, polymorphism, litter size traits, missense mutation

## Abstract

The newborn ovary homeobox gene (*NOBOX*) regulates ovarian and early oocyte development, and thus plays an essential role in reproduction. In this study, the mRNA expression level and single nucleotide polymorphism (SNP) of *NOBOX* in various tissues of Xiangsu pigs were studied to explore the relationship between its polymorphism and litter size traits. Also, bioinformatics was used to evaluate the effects of missense substitutions on protein structure and function. The results revealed that *NOBOX* is preferentially expressed in the ovary. Six mutations were detected in the *NOBOX* sequence, including g.1624 T>C, g.1858 G>A, g.2770 G>A, g.2821 A>G, g.5659 A>G, and g.6025 T>A, of which g.1858 G>A was a missense mutation. However, only g.1858 G>A, g.5659 A>G, and g.6025 T>A were significantly associated with litter size traits (*p* < 0.05). Further prediction of the effect of the missense mutation g.1858 G>A on protein function revealed that p.V82M is a non-conservative mutation that significantly reduces protein stability and thus alters protein function. Overall, these findings suggest that *NOBOX* polymorphism is closely related to the litter size of Xiangsu pigs, which may provide new insights into pig breeding.

## 1 Introduction

Litter size is one of the most important reproductive traits in sows that directly affect economic efficiency (1). It is a low heritability trait that is affected by many factors, such as genetics, environment, management, and nutrition (2). To improve economic efficiency, priority should be given to improving the reproductive quality of sows. However, relying on traditional crossbreeding selection techniques for improvement is significantly limiting. Given that SNPs in genes and trait association analysis methods are not affected by the environment, they are more efficient and accurate for the seed selection and expansion of high propagation populations (3, 4).

The newborn ovary homeobox (*NOBOX*) gene, an oocyte-specific homeobox gene, transcriptionally regulates oocyte-specific genes that play key roles in ovarian development, early oogenesis, and fertility (5–7). Research on the *NOBOX* gene has focused on its role in ovarian development and oogenesis. It is one of the most highly mutated genes in women with premature ovarian failure (8). Besides, *NOBOX* hypohydroxymethylation leads to ovarian dysfunction in offspring adult rats (9). At the same time, compound heterozygous truncating mutations in *NOBOX* characterized by double allele deletion mutations cause severe primary premature ovarian insufficiency (POI) with primary amenorrhea in patients in consanguineous marriages (10).

Pure heterozygous *NOBOX* truncation variants induce defective transcriptional activation, leading to primary ovarian insufficiency (11). Of note, immunolocalization, protein imprinting, and transcriptional assay have validated *NOBOX* mutations as the plausible causes of POI pathogenesis in HEK293T and CHO cells (12). *NOBOX* is also essential for signaling between somatic and germ cells during mouse embryonic development (13). In livestock, *NOBOX* is an essential maternal transcription factor during early bovine embryogenesis, thereby regulating embryonic genome activation, pluripotency gene expression, and blastocyst cell allocation (14). In zebrafish, *NOBOX* deletion does not affect testicular development and spermatogenesis; however, it plays an important role in ovarian differentiation and early follicular development. This suggests that *NOBOX* is closely related to reproduction (15).

The Xiangsu pig is a new pig line developed via crossbreeding after obtaining desirable production traits by backcrossing multiple Guizhou Congjiang Xiang pig (female parent) and Sutai pig (sire) generations with the Congjiang Xiang pig (sire) (16). This new line is characterized by early sexual maturity and strong disease resistance. In addition, it bears the high adaptability of the Congjiang Xiang pig and the high litter size and fast growth rate of the Sutai pig. In this study, we report for the first time six SNPs in the *NOBOX* gene of the Xiangsu pig. *NOBOX* function was predicted using bioinformatic techniques, and the correlation between the six SNPs and litter size was investigated. These results may guide the screening of candidate genes for sow reproductive performance, thereby benefiting the Xiangsu pig breeding program.

## 2 Materials and methods

### 2.1 Experimental animals

The animal experiments used in this study strictly complied with the guidelines of the Animal Welfare Committee of Guizhou University (EAE-GZU-2023-E015). A total of 142 healthy Xiangsu breeding sows under uniform feeding conditions were selected from the Guizhou University pig farm. The litter size traits, including the total number born (TNB), number born alive (NBA), and weaned piglet number were recorded per sow for the first and second litters (Supplementary material). Subsequently, blood samples were collected from the anterior vena cava of 142 pigs using a 5 mL EDTA anticoagulant tube and stored at −20°C. After the data collection of farrowing traits, three sows were randomly selected for slaughter, and their heart, liver, spleen, lung, kidney, longissimus dorsi muscle, and ovary tissue samples were collected and stored at −80°C in a refrigerator.

### 2.2 Extraction of whole blood DNA and tissue RNA

Genomic DNA was extracted from the anterior vena cava blood samples using a DNA extraction kit (DP348; Tiangen, Beijing, China) following the manufacturer’s guidelines. In addition, the total RNA was extracted from the heart, liver, spleen, lung, kidney, longissimus dorsi muscle, and ovary tissue samples using the TRIzol extraction kit (9109, TaKaRa, Dalian, China). The first strand of cDNA was synthesized via reverse transcription using a reverse transcription kit (A230; Kangrun, Beijing, China) according to the manufacturer’s interactions.

### 2.3 Primer design

The primers for DNA and cDNA amplification were designed according to the pig *NOBOX* DNA (accession number: NC_010451.4) and RNA (accession number: NM_001195116.1) sequences published on NCBI using Primer Premier 5.0 software (PREMIER Biosoft International, Palo Alto, CA, United States). Primers were synthesized by Qingdao Biotechnology Co., Ltd. (Chongqing, China), and the primer information is shown in Supplementary Table S1.

### 2.4 PCR amplification and real-time fluorescent quantitative PCR analysis

The PCR amplification of the genomic DNA was performed in a total volume of 20 μL, including 10 μL of 2× Taq PCR Master Mix (GeneStar, Beijing, China), 1 μL of forward primer, 1 μL of reverse primer, 1 μL of genomic DNA, and 7 μL of double-distilled water (ddH2O). The amplification conditions consisted of 35 cycles of 3 min of pre-denaturation at 94°C, 30 s of denaturation at 94°C, 30 s of denaturation at 63°C, 72°C annealing for 1 min, 72°C extension for 5 min, and preservation at 4°C. Next, 5 μL of amplification product was aspirated and subjected to 1% agarose gel electrophoresis for 25 min. The target bands were verified using a gel imaging system, and the amplicons were sent to Qingdao Biotech (Chongqing, China) for sequencing. The cDNA was amplified in a 20 μL q-PCR reaction system consisting of 1 μL of cDNA template, 0.5 μL of forward primer, 0.5 μL of reverse primer, 10 μL of 2× RealStar Fast SYBR qPCR Mix (GeneStar, Beijing, China), and 8 μL of ddH_2_O. The cDNA amplification was replicated four times. The reaction conditions were pre-denaturation at 95°C for 2 min, denaturation at 95°C for 15 s, annealing at 60°C for 30 s, and extension at 72°C for 30 s for 40 cycles. GAPDH was the fluorescence quantitative reference gene.

### 2.5 Bioinformatics analysis

*NOBOX* amino acid sequences from eight species, including *Homo sapiens* (XP_016867231.1), *Mus musculus* (NP_570939.1), *Equus caballus* (XP_ 023494578.1), *Sus scrofa* (NP_001182045.1), *Ovis aries* (XP_042104903.1), *Bos taurus* (XP_024846980.1), *Cervus elaphus* (XP_043727747.1), and *Gallus gallus* (XP_040516952.1) were obtained from NCBI,^1^ and a phylogenetic tree was constructed using MEGA7^2^ (17). Subsequently, the common motifs in the super secondary structure were predicted using the MEME tool^3^ to reveal the structural characteristics and functions of *NOBOX* proteins in the selected eight species (18). Besides, the multiple sequence comparison of the amino acid sequences of *NOBOX* was performed using Clustal Omega^4^ (19), and the results were uploaded to the online server Con Surf^5^ for sequence conservation evaluation. For the functional and stability studies of *NOBOX* proteins, PhD-SNP,^6^ SNAP2,^7^ I-Mutant2.0,^8^ and MuPro^9^ were used for prediction analysis (20–23). Additionally, Sopma^10^ and AlphaFold2 were used to predict the secondary structure and construct the 3D model of the tertiary structure of the protein (24, 25).

### 2.6 Statistical analysis

The presence of SNPs in *NOBOX* sequence was determined via peak plotting against the PCR sequencing reads using the SeqMan software (26). Wild-type and mutant sequences were aligned and compared using MegAlign and ClustalW software in the DNA Star package. Genotype and gene frequencies at each mutation locus were calculated directly. Hardy–Weinberg equilibrium (HWE) was evaluated using the chi-squared (*χ*^2^) test, and the gene polymorphism parameters included homozygosity (Ho), heterozygosity (He), number of effective alleles (Ne), and polypeptide information content (PIC) (27). Linkage disequilibrium (LD) and haplotype analyses among SNPs were performed using the SHEsis Main^11^ software (28), and the degree of chain imbalance was evaluated using the *r*^2^ value, where *r*^2^ > 0.33 indicated a strong chain imbalance state (29). On the other hand, diplotypes were evaluated based on haplotypes.

The difference in the number of litters between the different genotype groups was compared using one-way analysis of variance in the average drop-down option in IBM SPSS Statistics 25 software. The following general linear model formula was used: *Y_ijk_* = *μ* + *G_i_* + *S_k_* + *A_j_* + *e_ijk_*, where *Y_ijk_* is the litter size and number of pigs weaned, μ is the mean, *G_i_* is the fixed effect of genotype, *S_k_* is the random effect of sire, *A_j_* is fixed effect of age, and *e_ijk_* is the residual effect. The results are presented as the mean ± standard error (30).

*NOBOX* expression levels at different mutation sites were calculated using the 2^−ΔΔCtd^ method (31), and its expression patterns were mapped using the GraphPad Prism 8 software. Data are expressed as mean ± standard deviation at two decimal places.

## 3 Results

### 3.1 Expression profile of *NOBOX* in different tissues

**Figure 1:**
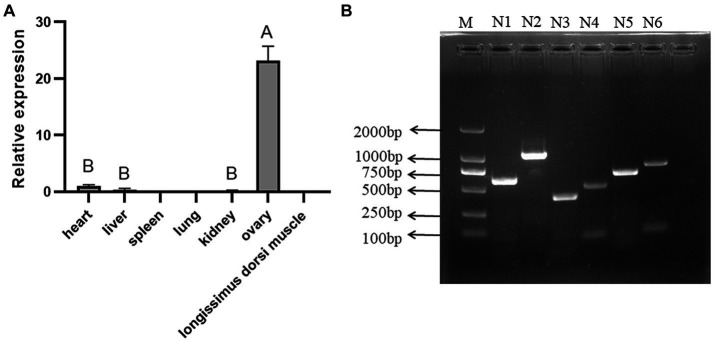
**(A)** Tissue expression of *NOBOX* in Xiangsu pig. Different capital letters indicated that the difference between different tissues was extremely significant (*p* < 0.01). **(B)** The results of gel electrophoresis imaging of PCR amplification products. M: DL2000 marker, N1–N6: *NOBOX* gene Exon1–Exon6.

The tissue expression profiles of *NOBOX* are shown in Figure 1A *NOBOX* was expressed in the heart, liver, kidney, and ovary, with the highest expression level in the ovarian tissues, which was significantly higher than that in the heart, liver, and kidney (*p* < 0.01). On the contrary, there were no significant differences in *NOBOX* expression levels among the heart, liver, and kidney (*p* > 0.05). In addition, *NOBOX* was not expressed in the spleen, lung, and longest dorsal muscle.

### 3.2 PCR gel electrophoresis imaging analysis

The gel electrophoresis imaging is shown in Figure 1B. The PCR amplification products were consistent with the target fragment size, with clear and single bands, non-specific amplification, and no obvious trailing phenomenon, implying the primers had good specificity.

### 3.3 Identification of *NOBOX* polymorphic loci

**Figure 2:**
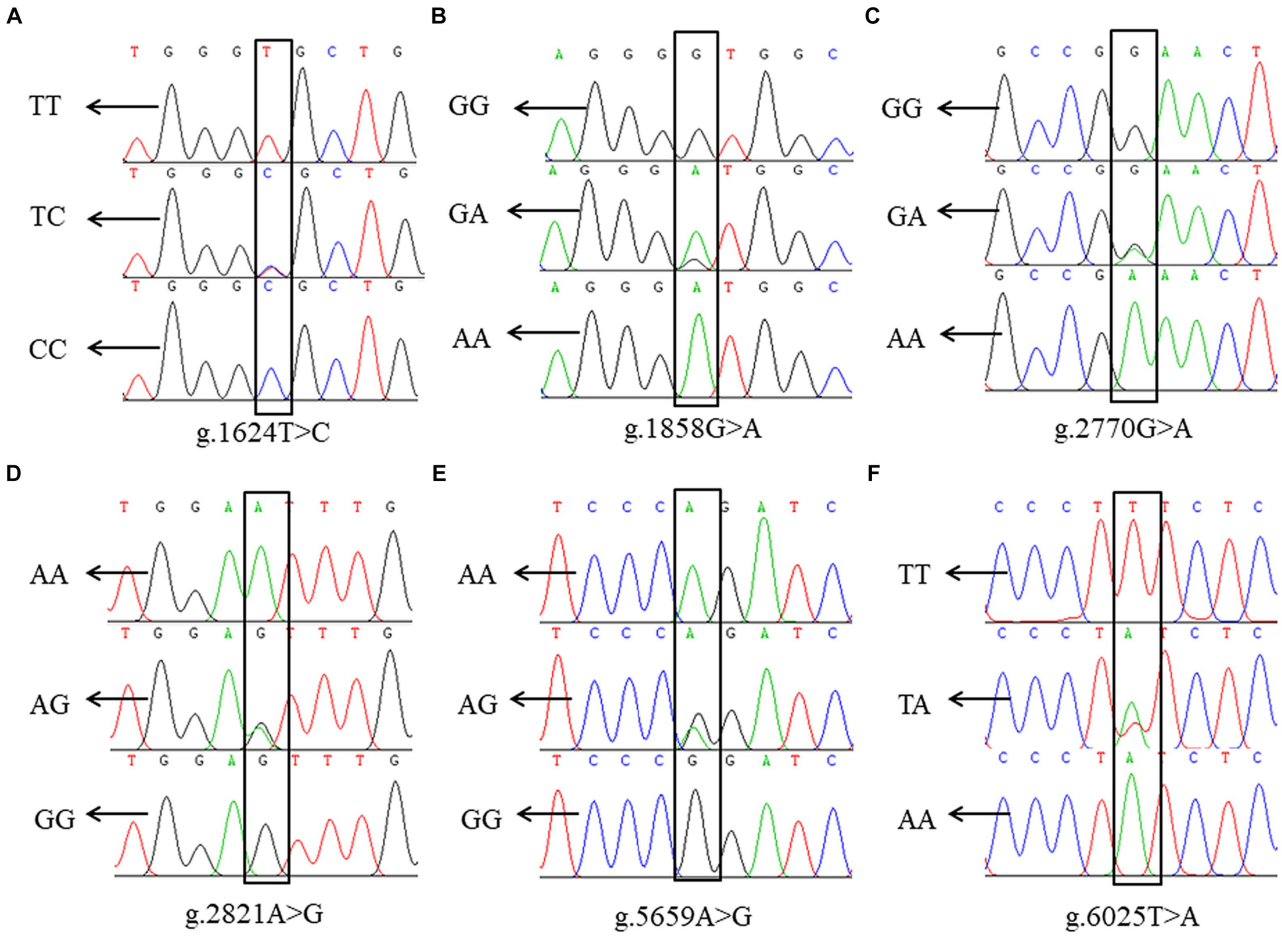
SNP locus of *NOBOX* in Xiangsu pig. **(A)** g.1624 T>C, **(B)** g.1858 G>A, **(C)** g.2770 G>A, **(D)** g.2821 A>G, **(E)** g.5659 A>G, **(F)** g.6025 T>A.

Sequence alignment between the sequencing results and the reference sequence of porcine *NOBOX* (Accession number: NC_010451.4) revealed six SNPs in *NOBOX*, which were labelled g.1624 T>C, g.1858 G>A, g.2770 G>A, g.2821 A>G, g.5659 A>G. Notably, two alleles and three genotypes were present in all the six SNPs (Figure 2). The wild-type and mutant sequence alignment revealed that the base G at the g.1858 G>A locus was mutated to A, altering the codon-GUG- to -AUG-. Consequently, methionine (M) replaced valine (V), and g.1858 G>A was a missense SNP. The mutation at g.5659 A>G changed the codon-CCA- to -CCG-, resulting in a synonymous mutation because-CCA- and-CCG- are simple codons and proline (P) was not replaced (Supplementary Figure S1).

### 3.4 Biological evolution and protection

**Figure 3:**
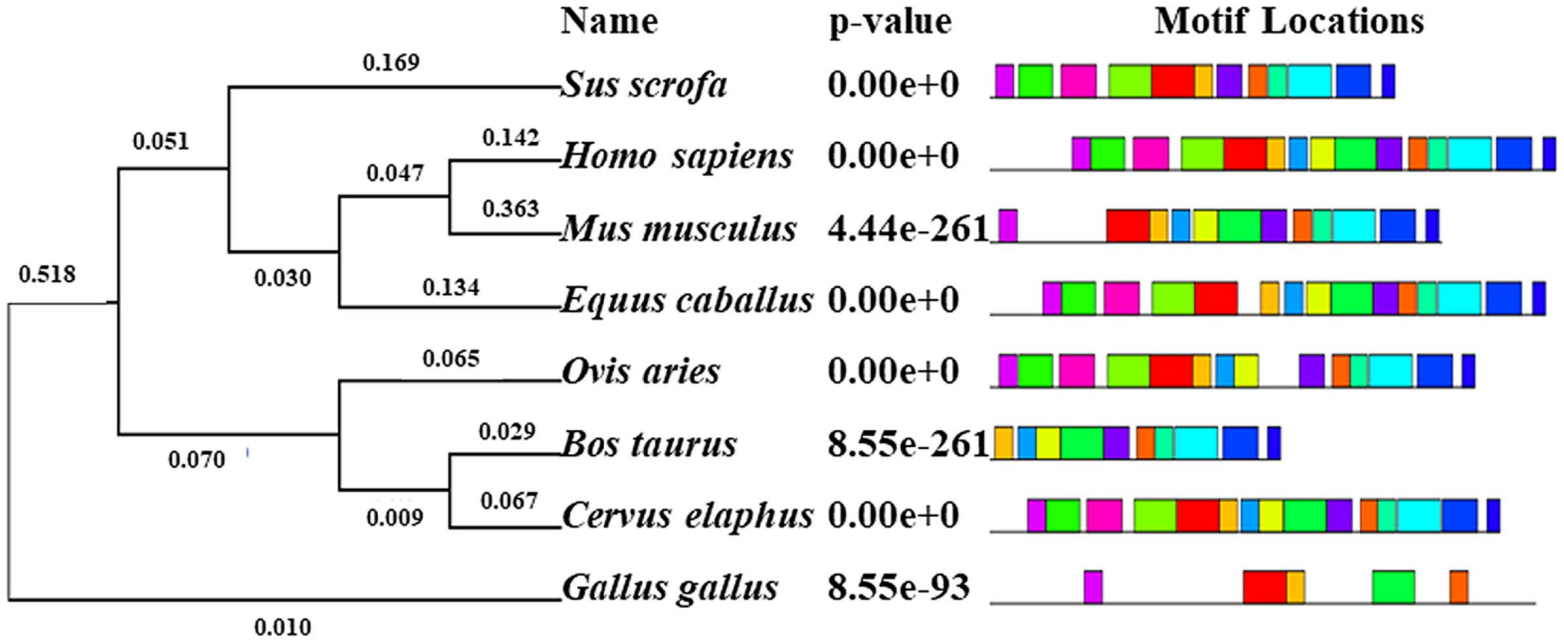
Phylogenetic tree (left) and motif structural analysis (right) for eight species. Fifteen significant motifs were identified. The length of the color block shows the position, strength and significance of a particular motif site. The length of the color block shows the position, strength and significance of a particular motif site. The length of the motif is proportional to the negative logarithm of the *p*-value of the motif site, truncated at the height for a *p*-value of 1 × 10^−10^. The colors were generated through motif analysis performed via the MEME suit system.

**Figure 4:**
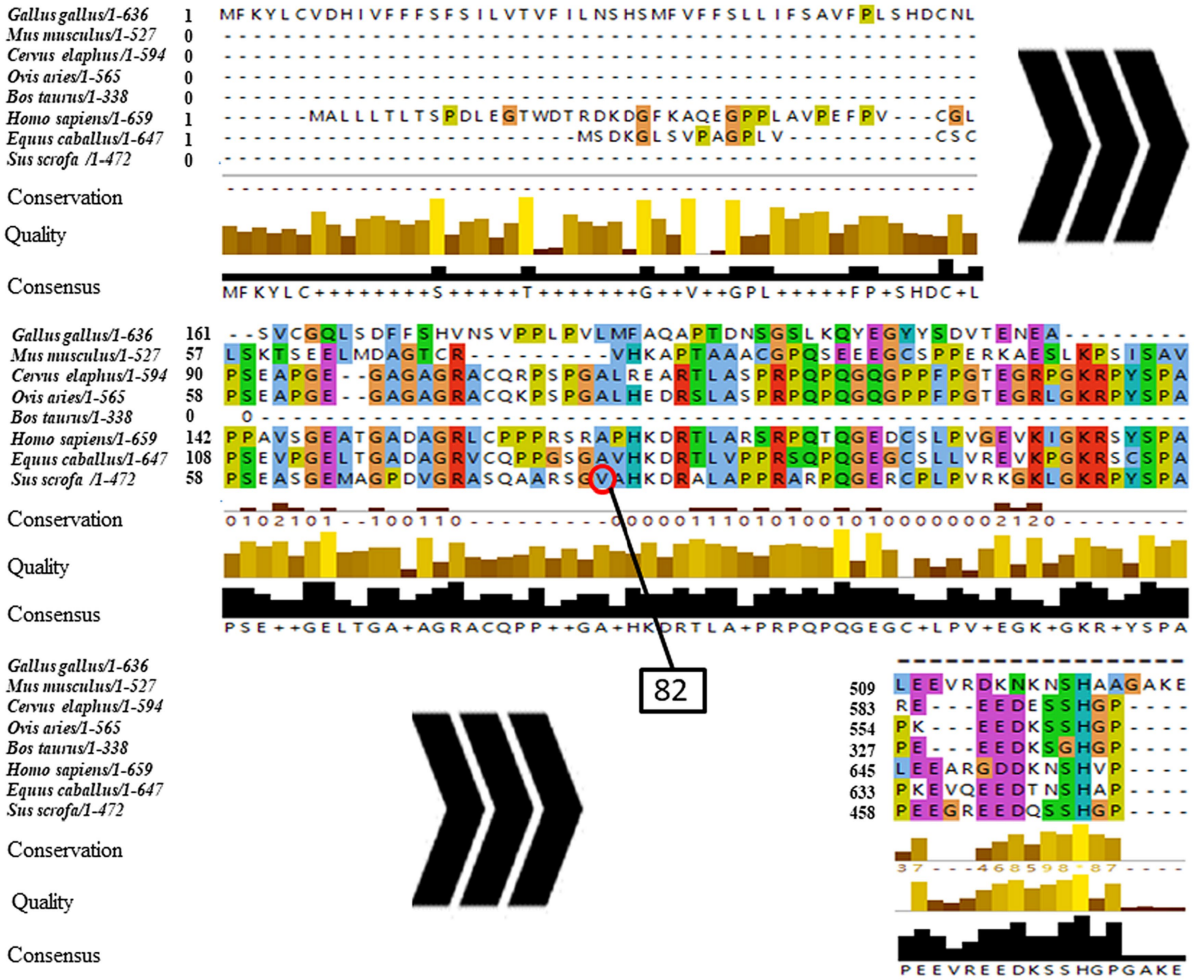
Conservative analysis of *NOBOX* SNP amino acid mutation sites. The red circle corresponds to p.V82M.

The phylogenetic tree of *NOBOX* sequences from the eight species is presented in Figure 3. Among the *NOBOX* species affinities, pig (*S. scrofa*) was most closely related to human (*H. sapiens*), followed by house mouse (*M. musculus*) and horse (*E. caballus*), and was furthest removed from the chicken (*G. gallus*). Fifteen significant amino acid sequences were detected in the eight species, indicating functional similarity at the super-secondary structure (Supplementary Figure S2). In addition, *NOBOX* was poorly conserved across species, with p.V82M as the nonconserved mutation (Figure 4).

### 3.5 Missense SNPs affect protein structure and function prediction

The predicted effect of missense SNPs on protein function based on the online prediction tool PhD-SNP yielded a score of 8, indicating a neutral effect. However, the prediction tool SNAP2 yielded a score of 37, suggesting altered protein function. Additionally, the prediction of protein stability using I-Mutant 2.0 and MuPro revealed the free energy changes of −1.21 and −0.53, respectively, with the P.V82M mutation reducing protein stability. A comparison of the secondary structure prediction results between the wild type and mutant revealed that p.V82M mutation increased the α helix and β turn and decreased the random coil (Supplementary Tables S2–S4).

**Figure 5:**
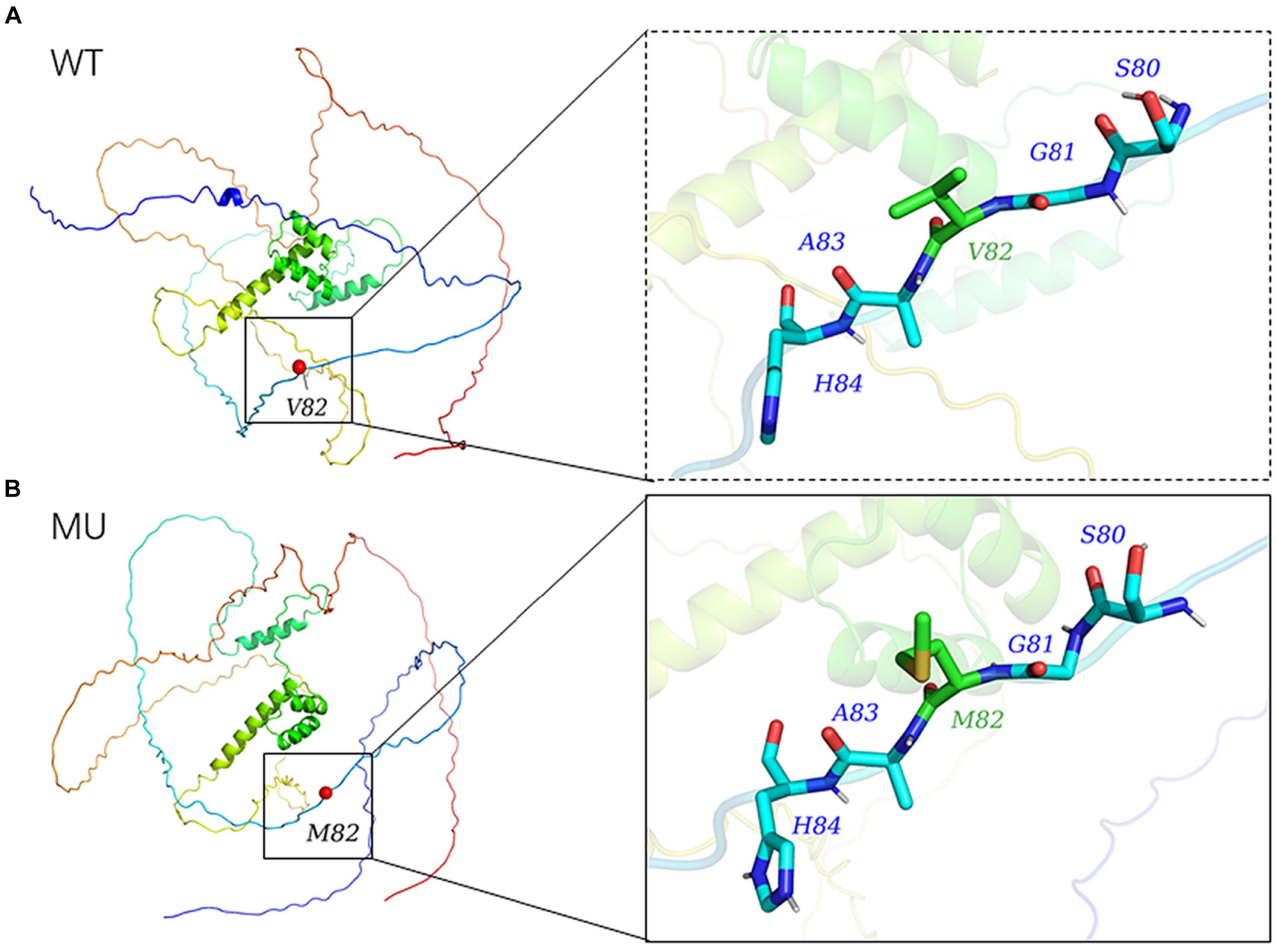
Modelled tertiary structure of the protein encoded by *NOBOX*. Different colors in the figure represent different secondary structures, **(A)** wild-type, **(B)** mutant type.

The 3D model of the *NOBOX* protein at p.V82M constructed using AlphaFold2 is presented in Figure 5. The p.V82M mutation replaced nonpolar, uncharged valine with a large, nonpolar, uncharged methionine, which altered the polar interactions with surrounding amino acids and affected the protein structure and function after the mutation.

### 3.6 *NOBOX* polymorphism in Xiangsu pig

According to *NOBOX* population genetic analyses (Table 1), the purity of each SNP locus was greater than the heterozygosity and the effective allele number ranged from 0.12 to 0.37. Besides, the polypeptide of SNP loci g.1624 T>C, g.1858 G>A, g.2821 A>G, g.5659 A>G, and g.6025 T>A was at the moderate polymorphism level (0.25 < PIC < 0.50). These five loci were in the Shangsu hybridization annotation, with strong selection potential and rich genetic diversity. The *χ*^2^ test revealed that all five SNP loci were in HWE (*p* > 0.05). However, the polypeptide content at locus g.2770 G>A was 0.12, a lower polymorphism level (PIC <0.25), contrary to the HWE based on the *χ*^2^ test (*p* < 0.05).

**Table 1:** Population genetic information of *NOBOX* SNPs.

SNP locus	Genotype frequency			Allele frequency		Ho	He	Ne	PIC	*χ* ^2^	*p*
g.1624 T>C	TT (79)	TC (53)	CC (10)	T	C	0.62	0.38	1.62	0.31	0.07	0.96
	0.56	0.37	0.07	0.74	0.26						
g.1858 G>A	GG (41)	GA (75)	AA (26)	G	A	0.51	0.49	1.98	0.37	0.66	0.72
	0.29	0.53	0.18	0.55	0.45						
g.2770 G>A	GG (125)	GA (14)	AA (3)	G	A	0.87	0.13	1.15	0.12	8.66	0.01
	0.88	0.10	0.02	0.93	0.07						
g.2821 A>G	AA (61)	AG (56)	GG (25)	A	G	0.53	0.47	1.88	0.36	3.50	0.17
	0.43	0.39	0.18	0.63	0.37						
g.5659 A>G	AA (69)	AG (57)	GG (16)	A	G	0.57	0.43	1.76	0.34	0.64	0.73
	0.49	0.40	0.11	0.69	0.31						
g.6025 T>A	TT (72)	TA (59)	AA (11)	T	A	0.59	0.41	1.69	0.33	0.05	0.98
	0.51	0.42	0.08	0.72	0.29						

Ho was homozygosity, He was heterozygosity, Ne was effective number of alleles, PIC was polymorphism information content. PIC <0.25 indicates low polymorphism, 0.25 < PIC < 0.50 represents moderate polymorphism, and PIC >0.5 denotes high polymorphism. *p* > 0.05 indicates that the gene frequency in the population is at Hardy–Weinberg equilibrium.

### 3.7 *NOBOX* LD and haplotype analyses

**Figure 6:**
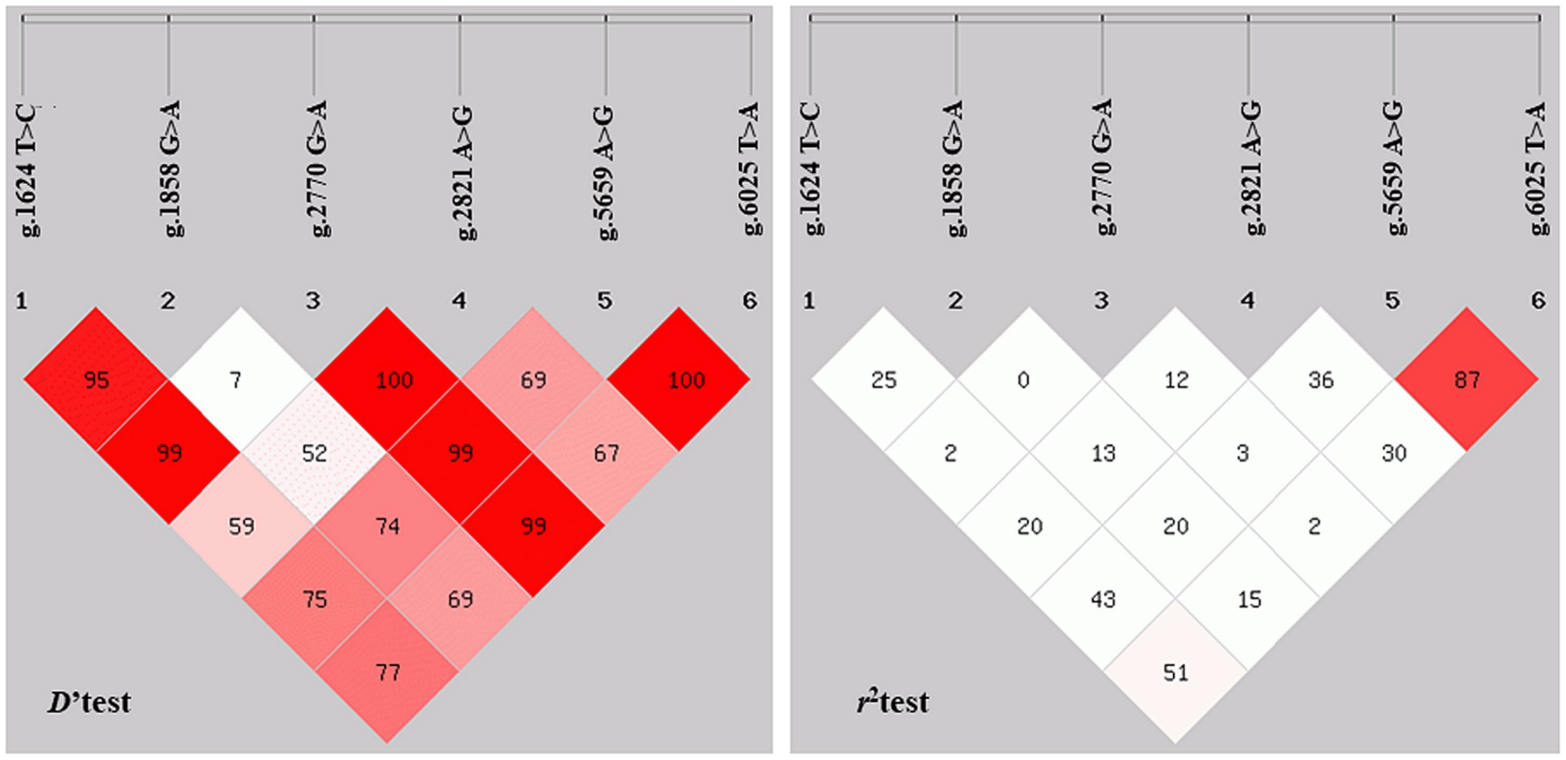
Analysis of linkage disequilibrium. *r*^2^ represents the correlation between a pair of loci, and *D*′ denotes the difference between the observed and the expected frequency of a given haplotype.

The LD analysis of *NOBOX* SNPs using *D*′ and *r*^2^ tests is shown in Figure 6 (32). LD analysis revealed that the *D*′ values ranged from 0.08 to 1.00 and *r*^2^ values from 0 to 0.874. SNP loci between g.1624 T>C and g.5659 A>G, g.1624 T>C and g.6025 T>A, g.2821 A>G and g.5659 A>G, and g.5659 A>G, and g.6025 T>A, with the *r*^2^ of 0.44, 0.52, 0.37, and 0.87, respectively, were n strong chain imbalance, with the strongest degree of chain imbalance between g.5659 A>G and g.6025 T>A (Table 2).

**Table 2:** Linkage disequilibrium coefficient between SNPs of *NOBOX*.

*D*′/*r*^2^	g.1624 T>C	g.1858 G>A	g.2770 G>A	g.2821 A>G	g.5659 A>G	g.6025 T>A
g.1624 T>C		0.26	0.03	0.20	0.44	0.52
g.1858 G>A	0.96		0.00	0.14	0.20	0.16
g.2770 G>A	0.99	0.08		0.13	0.03	0.03
g.2821 A>G	0.59	0.53	1.00		0.37	0.31
g.5659 A>G	0.76	0.74	0.99	0.69		0.87
g.6025 T>A	0.77	0.70	0.99	0.67	1.00	

Haplotype analysis identified three dominant haplotypes with frequencies greater than 5% from the Xiangsu pig population. They included Hap1 (TAGAAT), Hap2 (TGGAAT), and Hap3 (CGGGGA), with the frequencies of 36.20, 18.30, and 18.10%, respectively (Table 3).

**Table 3:** Haplotype analysis and frequency of *NOBOX* SNPs.

Haplotype	g.1624 T>C	g.1858 G>A	g.2770 G>A	g.2821 A>G	g.5659 A>G	g.6025 T>A	Frequency
Hap1	T	A	G	A	A	T	36.20%
Hap2	T	G	G	A	A	T	18.30%
Hap3	C	G	G	G	G	A	18.10%

Haplotypes with frequencies greater than 5.00% were selected, Hap1, TAGAAT; Hap2, TGGAAT; Hap3, CGGGGA.

### 3.8 Association analysis between *NOBOX* polymorphism and litter size traits in Xiangsu pigs

The correlation between the SNPs of pig *NOBOX* and litter size traits is shown in Table 4. The GG genotypes were significantly higher than AA genotypes at the g.1858 G>A locus and GG genotypes were significantly higher than AG genotypes at the g.5659 A>G locus in first-born TNB and NBA (*p* < 0.05). For g.6025 T>A, the TT and TA genotypes were significantly higher than AA genotype in the TNB and NBA of second-born sows (*p* < 0.05).

**Table 4:** Correlation analysis of *NOBOX* polymorphisms with litter size traits.

SNPs	Genotype	TNB^*^	NBA^*^	Number of weaned piglets^*^	TNB^**^	NBA^**^	Number of weaned piglets^**^
g.1624 T>C	TT (79)	9.59 ± 1.55	9.37 ± 1.42	9.19 ± 1.29	9.96 ± 1.43	9.71 ± 1.34	9.38 ± 1.10
	TC (53)	9.68 ± 1.57	9.53 ± 1.32	9.30 ± 1.23	9.85 ± 1.46	9.64 ± 1.33	9.43 ± 1.14
	CC (10)	10.20 ± 1.23	9.90 ± 0.99	9.20 ± 1.14	9.10 ± 0.74	9.00 ± 0.67	8.70 ± 0.67
g.1858 G>A	GG (41)	10.02 ± 1.59a	9.78 ± 1.29a	9.49 ± 1.27	10.00 ± 1.69	9.71 ± 1.42	9.41 ± 1.20
	GA (75)	9.61 ± 1.35ab	9.45 ± 1.21ab	9.20 ± 1.09	9.81 ± 1.40	9.64 ± 1.38	9.33 ± 1.11
	AA (26)	9.27 ± 1.85b	9.00 ± 1.74b	8.92 ± 1.60	9.77 ± 0.91	9.50 ± 0.86	9.31 ± 0.93
g.2770 G>A	GG (125)	9.72 ± 1.54	9.52 ± 1.34	9.30 ± 1.27	9.86 ± 1.44	9.64 ± 1.32	9.38 ± 1.12
	TA (14)	9.21 ± 1.48	9.00 ± 1.57	8.64 ± 1.08	10.00 ± 1.30	9.79 ± 1.19	9.36 ± 0.93
	AA (3)	9.67 ± 1.53	9.33 ± 1.15	9.00 ± 1.00	9.33 ± 1.15	8.67 ± 1.15	8.33 ± 0.58
g.2821 A>G	AA (61)	9.77 ± 1.61	9.52 ± 1.40	9.38 ± 1.32	9.95 ± 1.53	9.74 ± 1.37	9.43 ± 1.07
	AG (56)	9.64 ± 1.49	9.46 ± 1.26	9.21 ± 1.19	9.89 ± 1.32	9.70 ± 1.28	9.45 ± 1.19
	GG (25)	9.48 ± 1.48	9.32 ± 1.52	8.92 ± 1.22	9.56 ± 1.33	9.24 ± 1.20	8.96 ± 0.89
g.5659 A>G	AA (69)	9.77 ± 1.44ab	9.57 ± 1.30ab	9.36 ± 1.16	9.88 ± 1.43	9.67 ± 1.31	9.39 ± 1.05
	AG (57)	9.39 ± 1.69b	9.18 ± 1.45b	9.02 ± 1.37	9.96 ± 1.46	9.72 ± 1.39	9.42 ± 1.21
	GG (16)	10.25 ± 1.18a	10.06 ± 1.06a	9.44 ± 1.15	9.38 ± 1.09	9.19 ± 0.91	8.94 ± 0.85
g.6025 T>A	TT (72)	9.76 ± 1.46	9.54 ± 1.31	9.35 ± 1.18	9.92 ± 1.43a	9.69 ± 1.31	9.43 ± 1.06a
	TA (59)	9.44 ± 1.65	9.27 ± 1.46	9.07 ± 1.35	9.95 ± 1.44a	9.69 ± 1.37	9.39 ± 1.17a
	AA (11)	10.27 ± 1.19	10.00 ± 1.00	9.36 ± 1.21	9.00 ± 0.77b	8.91 ± 0.70	8.64 ± 0.67b

Different lowercase letters indicate significant differences (*p* < 0.05), same small letter differences are not significant (*p* > 0.05). TNB is the number of births, NBA is the number of live births. ^*^Indicates first litter performance per sow; ^**^indicates the litter performance of the second litter per sow.

The association analysis between diplotype and litter size traits is summarized in Table 5. The TNB and number of weaned piglets in the first-born sows were significantly higher in diploid Hap1/1 (TTAAGGAAAATT) and Hap1/3 (TCGAGGAGAGTA) than in Hap1/2 (TTGAGGAAAATT) (*p* < 0.05). Diplotypes with <5.0% frequency were not considered.

**Table 5:** Correlation analysis diplotype and litter size traits in Xiangsu pig.

Diplotype	TNB^*^	NBA^*^	Number of weaned piglets^*^	TNB^**^	NBA^**^	Number of weaned piglets^**^
Hap1/1 (17)	10.06 ± 1.64a	9.76 ± 1.39	9.71 ± 1.31a	9.82 ± 1.07	9.71 ± 0.92	9.65 ± 0.93
Hap1/2 (24)	9.17 ± 1.27b	9.04 ± 1.20	8.88 ± 1.12b	9.71 ± 1.49	9.46 ± 1.53	9.04 ± 1.04
Hap1/3 (26)	9.77 ± 1.18a	9.65 ± 1.02	9.35 ± 0.94a	9.85 ± 1.43	9.65 ± 1.44	9.46 ± 1.27

Hap1/1, TTAAGGAAAATT; Hap1/2, TTGAGGAAAATT; and Hap1/3, TCGAGGAGAGTA. Different lowercase letters indicate significant differences (*p* < 0.05), same small letter differences are not significant (*p* > 0.05). TNB is the number of births, NBA is the number of live births. ^*^Indicates first litter performance per sow; ^**^indicates the litter performance of the second litter per sow.

## 4 Discussion

Guizhou is rich in genomically pure and small pig breeds, including the Congjiang Xiang pig, one of the most famous small pig breeds (33, 34). These small pig breeds have the disadvantages of small size and low farrowing rate, which seriously hinders the economic development of local pig farming (35). However, compared with the Congjiang Xiangsu pig, the Xiangsu pig has the advantages of delicious quality meat and strong disease resistance, with greatly improved body size and reproductive performance. Therefore, Xiangsu pig line breeding significantly promotes the overall pig breeding and economic development in Guizhou.

*NOBOX* is important for promoting ovarian differentiation and development, regulating early oogenesis to mature female follicles, and regulating germ cell development (36, 37). It is also a key factor in the development of various germ cells and the main regulator of key oocyte genes and is closely related to the number and quality of mature follicles produced by women (38, 39). Based on this, it is valuable to establish the relationship between *NOBOX* mutations and female reproductive performance. However, there are no relevant studies on this relationship. More importantly, it is of research significance to explore the correlation between the variation of SNPs in *NOBOX* and litter performance in Xiangsu pigs.

The differences in gene expression among different tissues are related to their corresponding functions. In this study, the qRT-PCR analysis in the different tissues of adult sows revealed different *NOBOX* expression levels in the heart, liver, kidney, and ovary tissues, with the highest expression in the ovary, consistent with previous reports (14, 40). In addition, the mutation at g.1858 G>A, a missense SNP, replaced valine (V) with methionine (M). Missense mutations can reduce protein stability and are associated with phenotype (41, 42). Herein, the missense mutation altered *NOBOX* protein structure and function, thereby greatly reducing the protein stability. In addition, the p.V82M mutation increased the α helix and β turn proportions but decreased the random coil, which altered the different protein components that regulate the protein function.

Predicting the *NOBOX* protein structure and function revealed that pigs and poultry had the farthest genetic relationship during species evolution. In addition, 15 significant amino acid sequences were found in eight species, implying that they had functional similarity at the super-secondary structure. However, *NOBOX* was less conserved in different species, with p.V82M, a non-conservative mutation. Further assessment of gene polymorphisms revealed that the mutant locus g.2770 G>A did not conform to the population HWE, contrary to the other five SNPs, which may be caused by long-term human intervention during breeding (43). An assessment of LD among the six loci revealed that the synonymous mutation g.5659A>G and the 3′-UTR mutation g.6025 T>A had a strong linkage relationship (*D*′ = 1.000, *r*^2^ = 0.874) with the strongest degree of interlocking, implying that the two SNPs may have a synergistic effect on sow litter size (44, 45).

The correlation between *NOBOX* polymorphisms and sow litter size revealed that the SNP loci g.1858 G>A, g.5659 A>G, and g.6025 T>A were significantly correlated with sow litter size traits. At the same time, the litter and weaned piglet sizes were significantly lower for diploid Hap1/2 than for Hap1/1 and Hap1/3. Intronic mutations mostly affect mRNA shearing and folding at the molecular level and do not directly affect phenotypic traits (46, 47). For example, two intronic *NOBOX* mutations in Chinese women with premature ovarian failure (POF) were not associated with the disease (48), consistent with the intronic mutations in the present study, where g.1624 T>C, g.2770 G>A, and g.2821 A>G were not significantly associated with sow litter size traits (49, 50). In the present study, the 3′-UTR mutation locus g.6025 T>A was significantly lower in the AA genotype, characterized by fewer litters and weaned piglets than in TT and TA genotypes in the second litter. Besides, there was a significant correlation between the number of live piglets produced in the first litter for the synonymous mutation loci g.5659 A>G. g.5659 A>G and g.6025 T>A that belong to a strong cascade, confirming that these two SNPs had a synergistic effect on the number of litters born. The mutation from GG to AA at the missense SNP locus g.1858 G>A was accompanied by a gradual decrease in litter size and number of live and weaned piglets born in the first and second litters, consistent with the *NOBOX* mutation that causes POF in females (12, 51). Therefore, *NOBOX* may be an important molecular co-marker gene associated with porcine reproductive performance, which is significant in future molecular breeding improvement efforts in pigs.

## 5 Conclusion

This study identified six new SNPs in the pig *NOBOX*, including g.1858 G>A, a missense SNP that alters the amino acid sequence structure. Additionally, g.1858 G>A, g.5659 A>G, and g.6025 T>A significantly correlated with the litter size traits. Hap1/1, a high yielding dominant diploid, had the highest and most stable litter size traits. At the same time, g.1858 G>A significantly reduced the protein stability and greatly affected protein function. The heterozygous and homozygous genotypes after g.1858 G>A mutation gradually decrease the litter performance; thus, *NOBOX* may be an important SNP molecular marker gene for improving the litter performance of sows.

## Data availability statement

The original contributions presented in the study are included in the article/Supplementary material, further inquiries can be directed to the corresponding author.

## Ethics statement

The animal study was approved by Animal Welfare Committee of Guizhou University (EAE-GZU-2023-E015). The study was conducted in accordance with the local legislation and institutional requirements.

## Author contributions

JH: Data curation, Methodology, Writing – original draft, Writing – review & editing. YR: Conceptualization, Supervision, Writing – review & editing. MX: Data curation, Methodology, Writing – review & editing. LD: Methodology, Software, Writing – review & editing. CJ: Methodology, Resources, Writing – review & editing. JL: Data curation, Resources, Writing – review & editing. JX: Investigation, Software, Writing – review & editing. XC: Methodology, Resources, Writing – review & editing. HX: Conceptualization, Funding acquisition, Supervision, Writing – review & editing.

## Footnotes

^1^ www.ncbi.nlm.nih.gov/protein

^2^ www.megasoftware.net

^3^ http://meme.nbcr.net/

^4^ https://www.ebi.ac.uk/Tools/msa/clustalo/

^5^ https://consurf.tau.ac.il/consurf-old.php

^6^ https://snps.biofold.org/PhD-SNP/PhD-SNP.html

^7^ https://rostlab.org/services/snap2web/

^8^ http://gpcr2.biocomp.unibo.it/cgi/predictors/I-Mutant2.0/I-Mutant2.0.cgi

^9^ http://mupro.proteomics.ics.uci.edu/

^10^ https://npsa-prabi.ibcp.fr/cgi-bin/npsa_automat.pl?page1/4npsa%20_sopma.html

^11^ http://analysis.bio-x.cn
