# Supplementary material for: Association between polymorphisms in NOBOX and litter size traits in Xiangsu pigs
Source: Front Vet Sci. 2024 Mar 8;11:1359312. doi: 10.3389/fvets.2024.1359312 (PMC10959092; doi:10.3389/fvets.2024.1359312)
Supplement: Supplementary file 1 [file Table_1.docx]

Supplementary Table 1. Amplification primers for *NOBOX* gene of Xiangsu pig.

| Primer Names | Amplified Regions | Amplification Length/bp | Primer Sequence (5′→3′) | Annealing temperature/°C |
| --- | --- | --- | --- | --- |
| NOBOX-Exon1 | Exon1 | 562 | F:CAGTTCACCTGGAAGGGCTCTTT  R:TGTAGAGAGATTCGAGCCCCAAC | 62 |
| NOBOX-Exon2 | Exon2 | 1077 | F:GCTGTGGGGTTACCTGATTTGAT  R:CCATAGCCTTGCAACTGAGTTTC | 61 |
| NOBOX-Exon3 | Exon3 | 378 | F:GGAAACTCAGTTGCAAGGCTATG  R:GTGAAAACAGGCAAGAACACAGC | 63 |
| NOBOX-Exon4 | Exon4 | 517 | F:CCATACCAACCTGACAGAGACAAAC  R:GGAGGACAGAATGAAGACTGTAGCT | 56 |
| NOBOX-Exon5 | Exon5 | 679 | F:ATCCAGGCTATTACAGCAGTAGAGC  R:GATTAGACCCCTAGTCTTGGAACCT | 62 |
| NOBOX-Exon6 | Exon6 | 820 | F:GCTCCATTTTGTGTCACTGTTACC  R:TGAGTGAGGAGACACGTAATGACA | 65 |
| NOBOX-qPCR | Exon | 121 | F:GTCGGAAAAAGACTCGAACCCT  R:ACATTCACTGTCTGGGCGATCT | 57 |
| GAPDH | Exon | 169 | F: TTGTGATGGGCGTGAACC  R: GTCTTCTGGGTGGCAGTGAT | 58 |
