# Supplementary material for: Association between polymorphisms in NOBOX and litter size traits in Xiangsu pigs
Source: Front Vet Sci. 2024 Mar 8;11:1359312. doi: 10.3389/fvets.2024.1359312 (PMC10959092; doi:10.3389/fvets.2024.1359312)
Supplement: Supplementary file 2 [file Table_2.docx]

Supplementary Table 2. Prediction of protein function affected by missense SNP.

| Prediction software | SNP locus | Amino acid mutation locus | Prediction results | Score |
| --- | --- | --- | --- | --- |
| PhD-SNP | g.1858 G>A | p.V82M | Neutral | 8（0-9） |
| SNAP2 |  |  | Effect | 37（-100-100） |

Note: A higher score indicates a greater impact on protein function.

Supplementary Table 3. Missense SNP affects protein stability prediction.

| Prediction software | SNP locus | Amino acid mutation locus | Free energy change (DDG)/(kJ▪mol^-1^ ) |
| --- | --- | --- | --- |
| I-Mutant 2.0 | g.1858 G>A | p.V82M | -1.21 |
| MuPro |  |  | -0.53 |

Note: delta delta G(DDG)>0 indicates enhanced protein stability, -0.5<G(DDG)<0 indicates a slight decrease in protein stability, and G(DDG)<-0.5 indicates a significant decrease in protein stability.

Supplementary Table 4. Missense SNP affects protein secondary structure prediction.

| Type | Alpha helix | Extended strand | Beta turn | Random coil |
| --- | --- | --- | --- | --- |
| NOBOX-Wild | 12.08% | 4.87% | 1.69% | 81.36% |
| NOBOX-Mut | 12.29% | 4.87% | 1.91% | 80.93% |
