# Supplementary material for: Association between polymorphisms in NOBOX and litter size traits in Xiangsu pigs
Source: Front Vet Sci. 2024 Mar 8;11:1359312. doi: 10.3389/fvets.2024.1359312 (PMC10959092; doi:10.3389/fvets.2024.1359312)
Supplement: Supplementary file 4 [file Image_1.pdf]

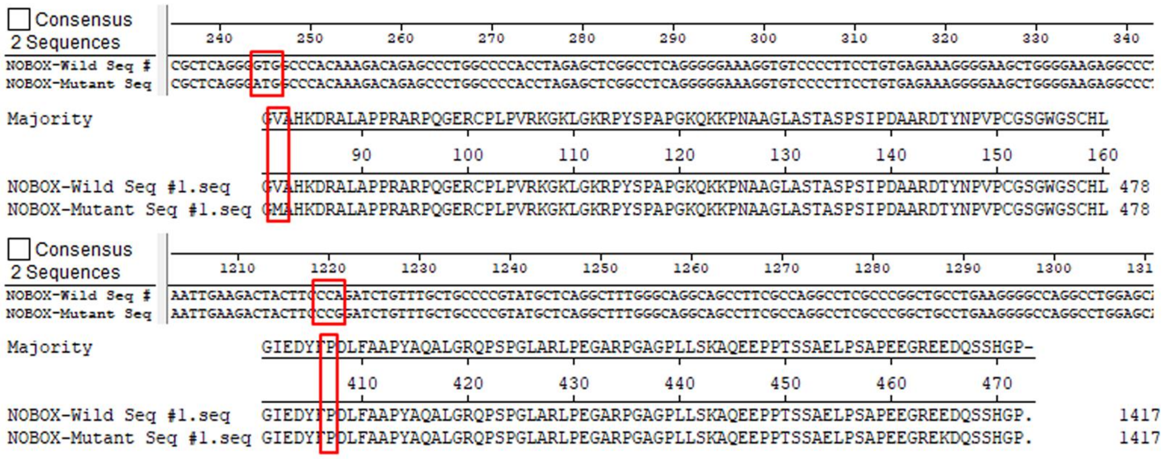

Supplementary Figure 1. Comparison of wild-type and mutant *NOBOX* amino acid sequences.The red frame region is the amino acid corresponding to the base.
